# Fly ash incorporated with biocement to improve strength of expansive soil

**DOI:** 10.1038/s41598-018-20921-0

**Published:** 2018-02-07

**Authors:** Mengmeng Li, Chaolin Fang, Satoru Kawasaki, Varenyam Achal

**Affiliations:** 1 Shanghai Key Lab for Urban Ecological Processes and Eco-Restoration (SHUES), School of Ecological and Environmental Sciences, East China Normal University, Shanghai, 200241 China; 2 Faculty of Engineering, Hokkaido University, Sapporo, 0608628 Japan; 3 Shanghai Institute of Pollution Control and Ecological Security, Shanghai, 200092 China

## Abstract

Microbially induced calcium carbonate precipitation (MICP) results in the formation of biocement (BC). This process, also known as biocementation, is recently widely used to improve the strength and durability of building materials including soils. In the present study, effectiveness of biocement as admixture with fly ash (FA) was investigated as first few studies to improve geotechnical properties of expansive soils. Biocement precipitated by *Bacillus megaterium* was blend with four formulations of fly ash at concentrations of 0, 10, 25 and 50%, namely 0% FABC, 10% FABC, 25% FABC, and 50% FABC, respectively. These formulations were separately added to expansive soils. Specimens with 25% FABC resulted in significant improvement in unconfined compressive strength of expansive soil that was more than two-times higher than control. Further, scanning electron microscopy-energy dispersive X-ray spectroscopy (SEM-EDX), Fourier-transform infrared spectroscopy (FTIR), and X-ray diffraction (XRD) analyses characterized microstructures of soil specimens, and depicted the process of MICP in improving strength of expansive soils. This research indicates that incorporation of biocement in fly ash is an effective means of increasing the strength of expansive soils.

## Introduction

Expansive soils are present throughout the world that have tendency to swell upon increase in moisture content. There is extensive damage caused by soil expansion reported from many countries worldwide. Such soils impose a potential risk to safety of civil engineering structures including highways, bridges, railways, airports, and seaports constructed on expansive soils^1^. When behavior of soils changes, it promotes severe land degradation. Thus, it is very important to stabilize expansive soils. Soil stabilization is the most common ground improvement technique that modifies soils to improve the engineering properties including strength of soils.

Additives are commonly used to improve the engineering or geotechnical properties of expansive soils using traditional stabilizers such as cement, gypsum, limestone, and industrial byproducts including fly ash. However, in order to reduce the cost and environmental impact due to secondary pollutant nature of some of these additives^2^, industrial byproducts are materials of choice by many researchers. Vast quantities of fly ash are produced as waste by utilization of coal in power generation that consists of spherical particles of a type of silicate^3^. Fly ash could even negatively impact soil texture and hydraulic properties of soils^4^, so it is very much crucial how to utilize this waste product. On the other hand, fly ash has been reported positively in soil-amendment agent, applications in agriculture, forestry, and land reclamation^5,6^. In addition, fly ash has shown its potential in a variety of construction applications^7,8^.

There are few reports where fly ash showed its potential in increasing the strength of expansive soil after adding in; however, strength is limited. Thus, in order to enhance the strength of such amendment, researchers are looking for various techniques. Microbially induced calcium carbonate precipitation (MICP) is one of recent environment friendly techniques used to improve the mechanical properties of porous materials^9–13^. It is a type of biomineralization that produce biocement, which is widely used to improve the durability of cementitious materials^14,15^. Such environmentally sound sustainable development is increasingly essential. This MICP process is dependent on ureolytic bacteria, which produce calcite and aragonite, the most common forms of calcium carbonates based on urease activity^16^. In order to make MICP process successful in improving strength of expansive soil while mixing with fly ash, it is very important for bacteria to survive in this formulation. Fly ash is used as a carrier material to enhance the metabolic activity of calcite-precipitating bacteria that supports survivality of bacteria for effective calcite production^8^. Due to this mechanism, fly ash together with bacterial calcium carbonate could enhance the strength of expansive soils.

Thus, in the present study a fixed amount of biocement precipitated from *Bacillus megaterium* added from lower to high concentrations of fly ash at 10, 25 and 50% was used as stabilizer to improve the engineering properties of expansive soils. All such soils were tested to obtain Atterberg limits, swelling potential, and unconfined compressive strength (UCS). Further, all specimens were characterized to evaluate potential of biocement in the enhancement of performance characteristics of expansive soils using SEM-EDX, FTIR, and XRD analyses. This is few of such detail studies carried out to improve the strength of expansive soils.

## Materials and Methods

### Materials

Expansive soil samples were collected from a slope along Wuhan-Xian expressway in Hubei Province, China. Fly ash used in this study was bought locally in Shanghai. The physicochemical characteristics of fly ash used are given in Table 1.

**Table 1:** Chemical composition of used fly ash.

Constituents	SiO_2_	Fe_2_O_3_	Al_2_O_3_	CaO	MgO	f-CaO	SO_3_	TiO_2_	LOI*
**Weight (%)**	60.98	6.7	24.47	4.9	0.68	0.58	0.52	< 0.1	1.86

*Loss of ignition.

### Biocement production

A urease producing *Bacillus megaterium* CGMCC 1.1741 grown in nutrient broth media containing substrate urea and calcium ion was used to produce biocement in the present study. The composition of media is provided in Achal *et al*.^17^. The grown bacterial precipitate was converted into lyophilized powder, termed as biocement^14^. Biocement was stored at −20 °C prior to use.

### Sample preparation

The expansive soil sample was dried in oven at 105 °C and then sieved to obtain a uniform distribution. Fly ash with amount of 0, 10, 25 and 50% by dry weight of soil were added into the soil. A fixed amount (5%) of biocement was mixed properly thereafter in all soil specimens containing various concentrations of fly ash to obtain a homogenous mixture. The final expansive soil specimens with biocement and 0, 10, 25, and 50% fly ash are coded as 0% FABC, 10% FABC, 25% FABC, and 50% FABC, respectively. The optimum water content to prepare specimens to test engineering properties of soil was obtained as per standard proctor compaction test^18^. Accordingly, maximum dry density values for each sample were calculated. All the specimens were tested after curing, with nutrient media containing urea and CaCl_2_, of 28 days, considering it standard time in testing building materials, to check results thereafter.

### Atterberg limits

Atterberg limits are important to measure properties and behavior of soils with respect to critical water contents that include liquid limit, plastic limit and plasticity index. These values from expansive soil specimens with stabilizers were calculated according to ASTM D 4318-00^19^.

### Free swell testing method

The free swell testing method was used to determine the swelling potential of the specimens^20^ using a mold of 70 mm diameter and 20 mm height. The specimens were statically compacted in oedometer rings after placing dry filter papers and air-dry porous stones on top and bottom of the specimen. The specimens were inundated with water under the nominal pressure of 6.25 kPa that lead to swelling of specimens. The experiments were terminated once the dial gauge readings became nearly constant.

The percent swell was determined by calculating constant (swell) value and final value (maximum deflection), according to following formula:

$$ \% \,{\rm{Swell}}\,{\rm{potential}}={\rm{dH}}/{{\rm{H}}}_{0}\times 100$$

where, dH = Increase in specimen height at given time interval; and H_0_ = Initial height of the compacted specimen.

### Unconfined Compressive Strength (UCS) test

To evaluate the shear strength parameters of all soil specimens, the unconfined compressive strength test was performed. The specimens were molded in thin stainless steel tubes of 76 mm height and 36 mm diameter and sheared at an axial strain rate of 1% per minute. The experiments were conducted in triplicate and average of the value was reported.

### SEM-EDX, FTIR and XRD analyses

The microstructures of all soil specimens (gold-coated) were examined under a S4800 (Hitachi, Japan) electron microscope operating at 200 kV, equipped with an energy dispersive X-ray (EDX) detector that detailed surface composition. FTIR spectra of soil specimens were recorded on a Thermo Nicolet Nexus 600 spectrophotometer (Thermo Nicolet, USA) at a wave band from 4000 to 400 cm^−1^ and presented from 4000 to 500 cm^−1^ using KBr pellets technique.

XRD was used to identify bio/minerals and other compounds of expansive soil specimens including those with specified amount of fly ash or fly ash and biocement using Ultima IV X-ray diffractometer (Rigaku, Japan) with CuKα radiation. Measurements were taken from 4 to 80°(2θ) during one hour. Crystalline phases of all specimens were identified using the database of the International Center for Diffraction Data.

## Results and Discussion

### Atterberg limits

**Figure 1:**
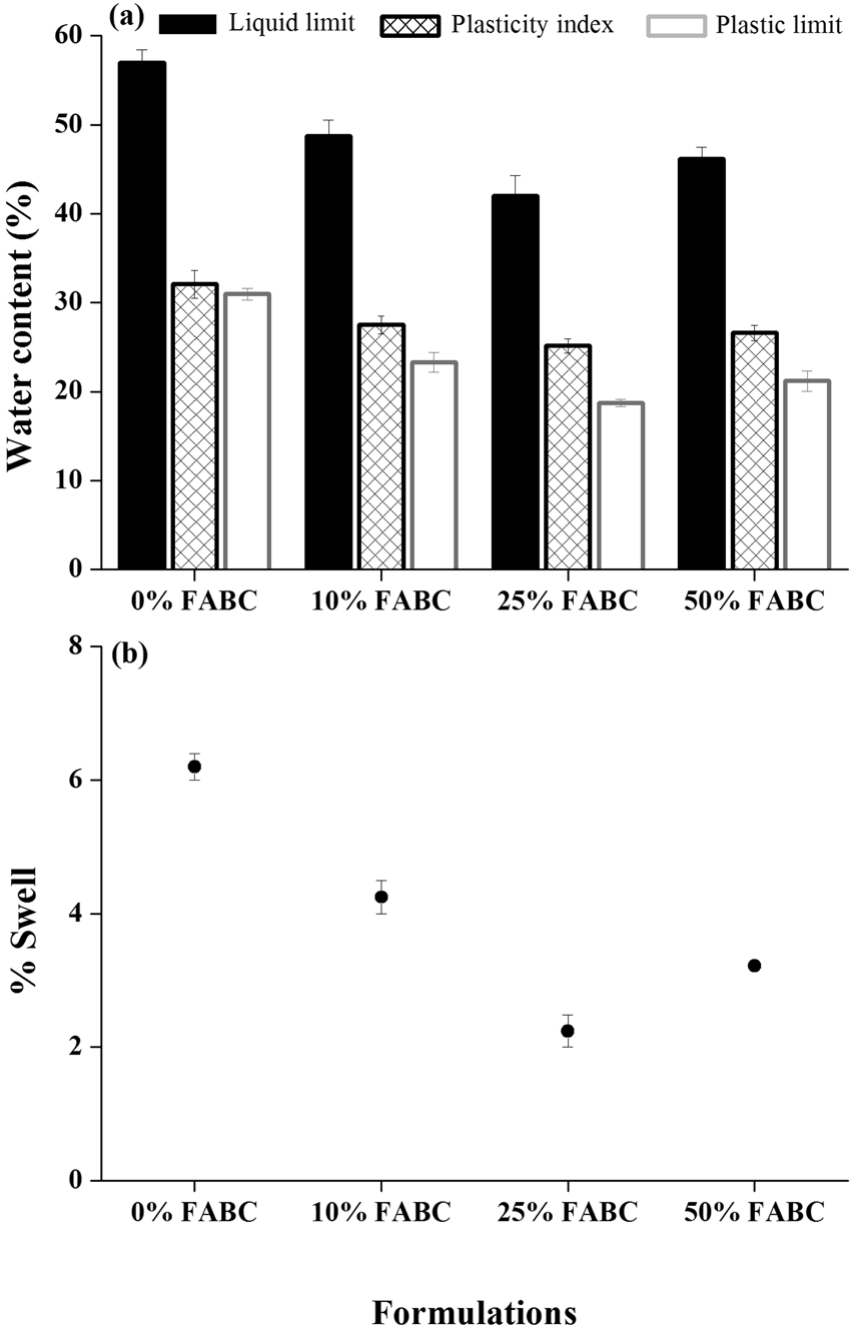
Variations of **(a)** Atterberg limits and (**b**) swelling potential, of expansive soils with different formulations of fly ash with biocement.

Behavior of soils in related to the amount of water in it for which Atterberg limits are calculated. The liquid limit values of the expansive soil decreased with an increasing amount of fly ash till 25% concentration and similar trends of plasticity index and plastic limit were observed (Fig. 1a). Plasticity index is a good indicator of swelling potential^2^. The activities of expansive soil were reduced greatly at 25% fly ash in the presence of biocement (25% FABC). Addition of fly ash together with biocement aids flocculation, and aggregate the clay particles that result in decrease in Atterberg limits of expansive soils.

### Swelling potential

Effect of different concentrations of fly ash with biocement on the swelling potential of the expansive soil is shown in Fig. 1b. The results showed that at a constant amount of bacterial carbonate, the swell potential for all the specimens decreased with an increase in the fly ash content; however, increased at 50% fly ash.

The higher swelling for the expandable soil in the absence of fly ash and bacterial carbonate can be attributed to the presence of mineral montmorillonite^21^. At certain concentrations, fly ash provides multivalent cations such as Ca^2+^, Fe^3+^, and Al^3+^, which may form minerals in the presence of biocement due to co-/precipitation. Such ions promote flocculation of clay particles by cation exchange resulting in decrease in the surface area and water affinity that ultimately reduces the swelling potential^2^. Further, bacterial calcite leads to formation of cementing compound, which was promoted in the presence of 10% and 25% fly ash due to favorable environment for bacterial growth; however, higher fly ash concentration (50%) may limit bacterial growth by reducing the bioavailability of nutrients. This process can effectively decrease the swelling potential. Fly ash is known to provide support for microbial activities in ground improvement^22^. It has been utilized as a carrier material for calcifying bacteria and has been proved that fly ash at some concentrations support bacterial metabolic activity to produce calcium carbonate^8^.

### Unconfined Compressive Strength (UCS)

**Figure 2:**
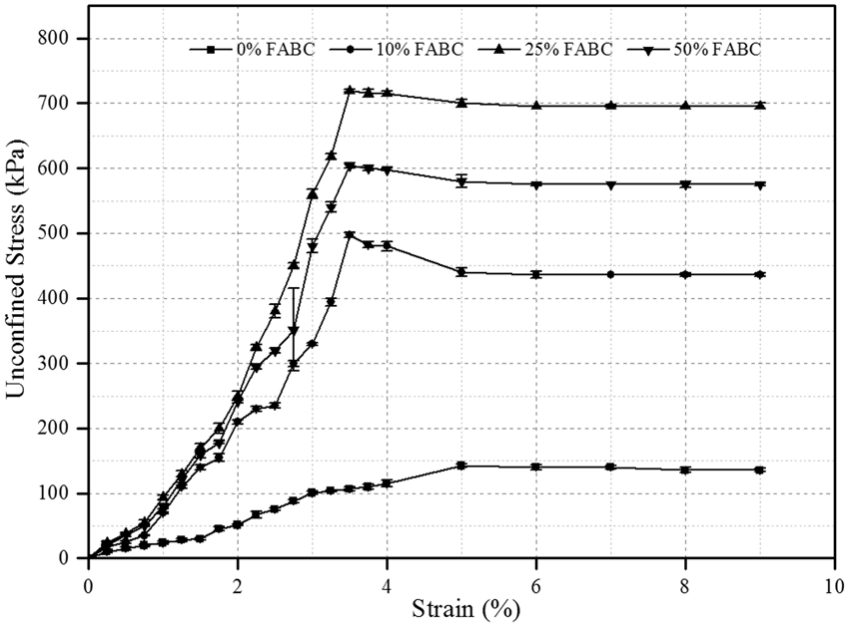
Stress shear strength versus strain for expansive soils with different formulations of fly ash with biocement.

The variation in unconfined compressive strength of expandable soil specimens with different concentrations of fly ash in the presence of biocement is shown in Fig. 2. Mere UCS of 142.6 kPa was obtained in 0% FABC expansive soils; however, significant increase in UCS value was measured in specimens of 10% FABC and 25% FABC, with maximum UCS of 719.4 kPa in 25% FABC specimen. Moreover, at this point the increase in strength can also be resulting from fly ash only. To find out if this maximum strength improvement in expansive soil containing 25% FABC was actually due to the presence of biocement in it, UCS test was additionally carried out for expansive soil containing only 25% FA. The UCS of specimens containing 25% FA was 294.6 kPa, which was significantly lower than those containing 25% FABC. Thus, the improvement in UCS in expansive soils with FABC is probably due to deposition of CaCO_3_ within the pores of soil-fly ash matrix, which plug the pores in the system. Soil (sand) grains are bound together by calcite crystals^16^. The effective fly ash content for the improvement of soil engineering properties reported to vary between 15 and 30%^23^.

At very high concentration of fly ash, that is 50% FABC, lower UCS was recorded than that of 25% FABC, which could be resulting from the reduction in efficiency of carbonate precipitation because of poor accessibility of bacterial cells due to entrapment in fly ash particles^7^. There is also possibility that high concentration of fly ash could decrease bacterial metabolic activity by reducing the bioavailability of nutrients, which leads to lesser precipitation. Fly ash, further, generates more amounts of hydration products coming from pozzolanic reaction^24^ that also increase the strength of expansive soils. In addition, it is known as a self-cementing agent (that property was enhanced in the presence of biocement) to increase the strength and to stabilize soil^22^.

### SEM-EDX

Examining the morphology of the expansive soil and soils with stabilizer, SEM demonstrated the MICP-based mechanism that leads to stabilization of the soil. In line with the aforementioned impact of MICP, crystalline mineral structure was observed in such specimens.

**Figure 3:**
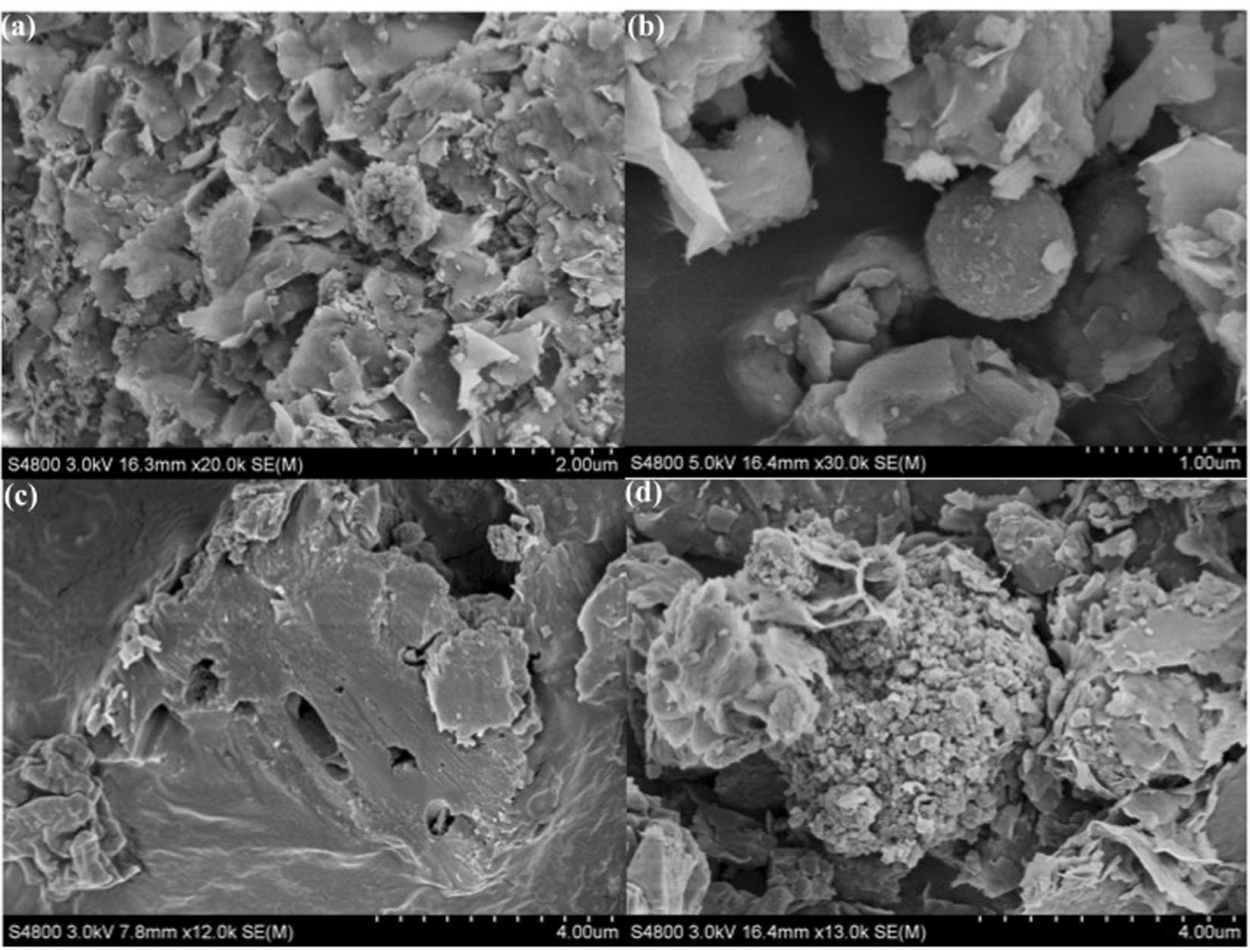
SEM micrographs of: (**a**) the natural expansive soil sample, (**b**) expansive soil with 10% FABC, (**c**) expansive soil with 25% FABC, and (**d**) expansive soil with 50% FABC. A typical image is shown from many similar examples.

Soil is very complex system where it is not easy to observe bacteria easily using SEM; however, holes embedded with rod-shaped bacterial cells were seen with crystal calcite structure in 25% FABC specimens (Fig. 3c). Some crystal type structures, which possibly could be calcium carbonate, were also observed in 10% and 50% FABC specimens (Fig. 3b and d). It could also be noted that as bacterial calcite contained bacterial cells, they grew or activated due to the presence of water and organic matter coming from soil and continued precipitation of carbonates, especially with specimen 25% FABC. The results were in agreement with Cheng *et al*.^25^ and Sham *et al*.^26^, where calcite-precipitating bacteria led to formation of different forms of calcium carbonate that enhanced UCS of soil. On other hand, natural expansive soil sample showed typical soil morphology (Fig. 3a), in agreement with Goodarzi *et al*.^27^.

**Figure 4:**
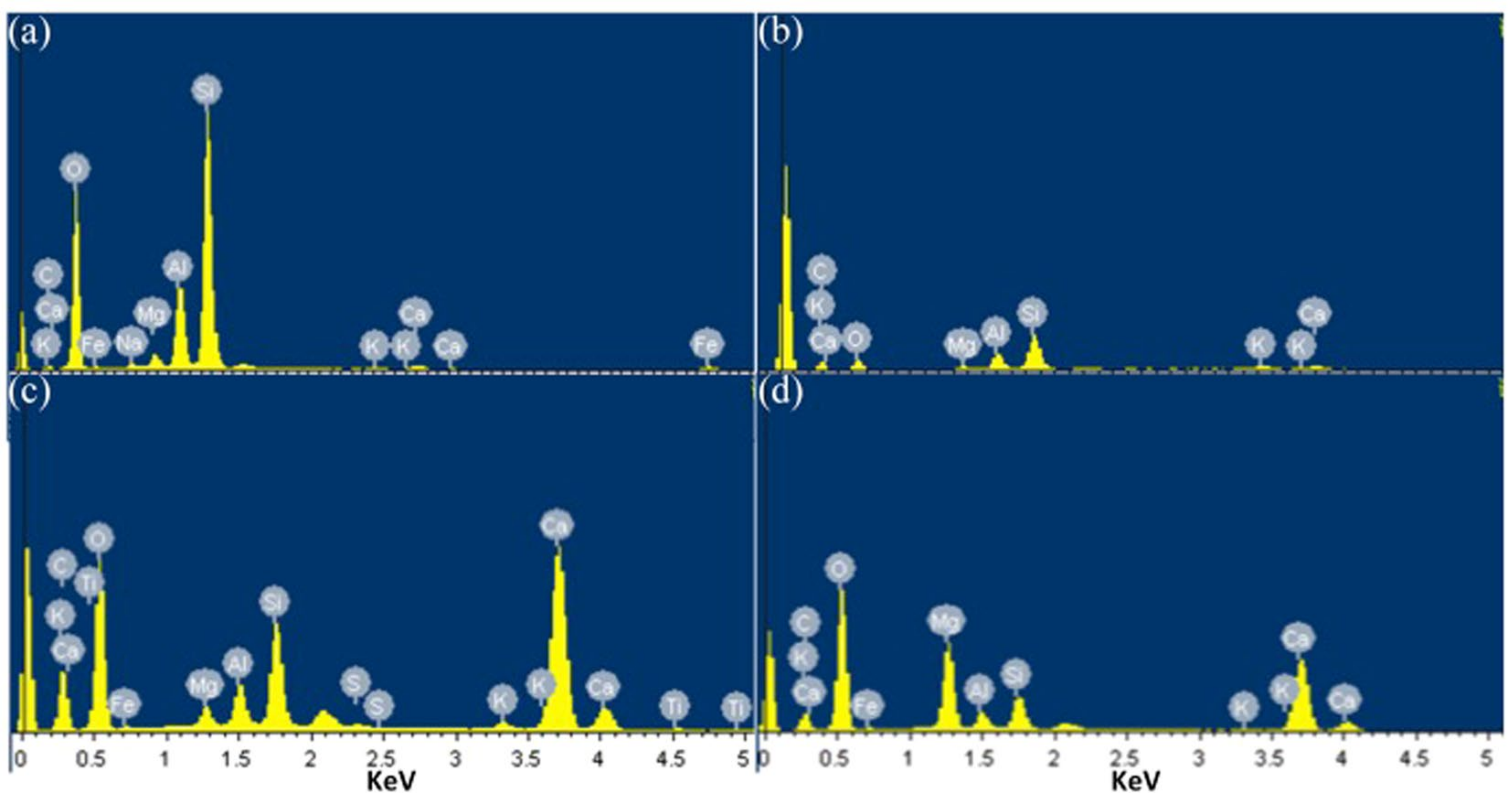
EDX spectra of: (**a**) the natural expansive soil sample, (**b**) expansive soil with 10% FABC, (**c**) expansive soil with 25% FABC, and (**d**) expansive soil with 50% FABC. EDX data are shown from one of several determinations.

Mineral constituents of expansive soil specimens with 0% FABC, 10% FABC, 25% FABC and 50% FABC were further characterized by EDX (Fig. 4). The spectra depicted more amount of CaCO_3_ formed in 25% FABC, followed by 50% FABC and 10% FABC. Although, based on this EDX analysis, the amount of calcium recorded were found to be 13.45%, 15%, 21% and 18%, respectively (data not provided); these values may vary due to data of semi-quantitative nature coming from EDX. Quantitative data obtained from EDX may not be reliable; however, together, SEM-EDX analysis provided direct and comprehensive information about the MICP process and change of surface compositions in various expansive soil specimens.

### FTIR and XRD

In order to confirm the MICP process leading to strength improvement in expansive soil specimens containing 25% FABC, samples were analyzed with FTIR and XRD, and results were also compared with expansive soil specimens containing 25% FA.

**Figure 5:**
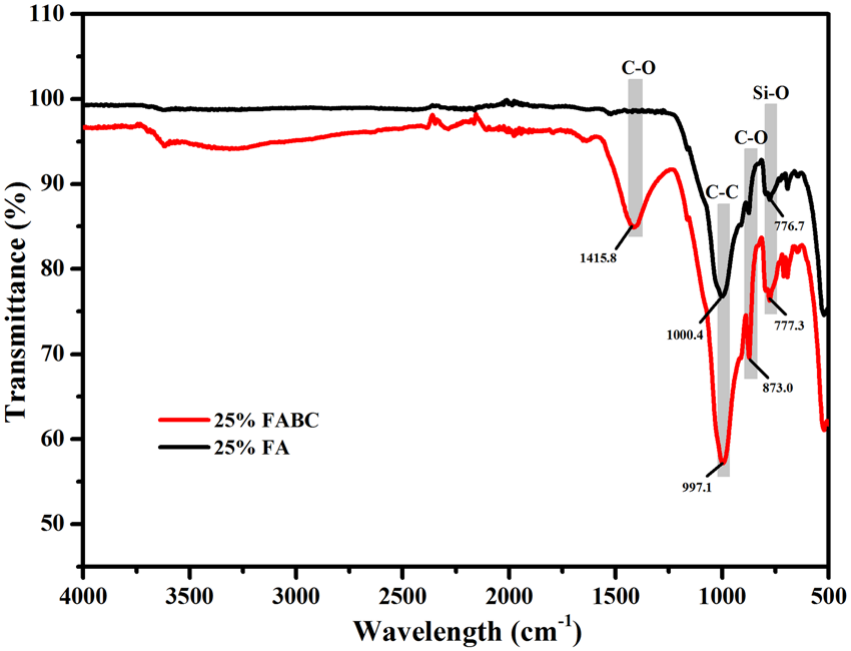
Comparative FTIR spectra of expansive soils containing 25% FA and 25% FABC. Typical data are shown from one of several determinations.

Strong absorption bands observed at 873 cm^−1^ and 1415 cm^−1^ that attributes to the characteristic C-O bonds in calcium carbonate were present mainly in 25% FABC soil specimen confirmed MICP process^14^ in the improvement of engineering properties of expansive soil. These characteristic peaks were absent in 25% FA specimen (Fig. 5). Absorption band at 777 cm^−1^ was found in both 25% FA and 25% FABC soil specimens could be assigned to Si-O stretching vibrations that is indicative of the presence of quartz in the expansive soil.

The main mineralogical constituents of expansive soil were quartz, montmorillonite, and illite with calcite as reflected under XRD pattern (Supplementary Figure 1). It is important to emphasize that expansive soil used for the experiment was not sterilized but was in its natural form; therefore, it may get contaminate with bacteria from other source. The similar mineral constituents were also identified in expansive soil by other researchers^28,29^. The XRD and EDX analyses showed that the expansive soil sample contained high amount of montmorillonite and its main exchangeable cation is sodium ions. Therefore, it can be considered as a Na^+^-dominant montmorillonite, which has a high tendency to swelling, in agreement with other studies^27,30^. The structure of montmorillonite breaks by addition of fly ash and calcium carbonate (produced here by biocementation)^31^.

**Figure 6:**
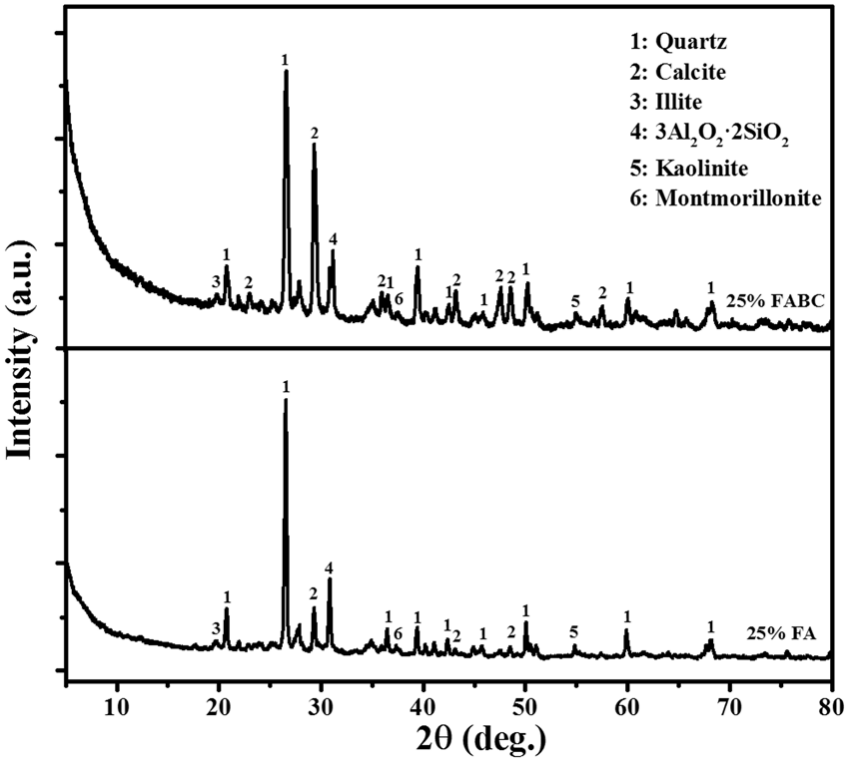
Comparative XRD patterns of expansive soils containing 25% FA and 25% FABC. Typical data are shown from one of several determinations.

XRD analysis of expansive soil specimens with 25% FA and 25% FABC revealed quartz as major constituents along with small peaks of illite, montmorillonite, and kaolinite (Fig. 6). Moreover, several calcite peaks were found in 25% FABC soil specimen. Comparatively, XRD patterns indicated lower peaks of calcite identified in specimens with 25% FA than specimen containing 25% FABC stabilizer (Fig. 6), denoting how biocement significantly contributed to calcite precipitation. XRD confirmed those crystals as calcites, consistent with that observed in SEM. The mechanism behind the formation of cementing compounds that binds granular particles of expansive soil to increase strength was due to MICP process where bacteria formed carbonates continuously.

## Conclusions

This research will promote utilization of fly ash combined with biocement for soil stabilization, which is mainly used for cement replacement in concrete. The results of present study recommend the use of 25% FABC for significant improvement in the strength of expansive soil. As bacteria can produce calcite continuously, it adds an important property for required reaction towards strength improvement of expansive soil. This is one of few studies where biocement was directly applied for any type of soil improvement. Future work is required to carry on expansive soil specimens of larger size, especially to study the MICP process in area of anaerobic zone. Together with biocement, fly ash would insure long-term ecological maintenance to enhance the performance characteristics of expansive soil.

## Electronic supplementary material

Supplementary information

## References

[1] Dang LC, Fatahi B, Khabbaz H (2016). Behaviour of expansive soils stabilized with hydrated lime and bagasse fibres. Procedia Engin..

[2] Cokca E (2001). Use of class C fly ashes for the stabilization of an expansive soil. J. Geotech. Geoenviron. Engin..

[3] Kumar D, Singh B (2003). The use of coal fly ash in sodic soil reclamation. Land Degrad. Develop..

[4] Pereira P (2014). Wildfire effects on extractable elements in ash from a *Pinus pinaster* forest in Portugal. Hydrolog. Process..

[5] Jala S, Goyal D (2006). Fly ash as a soil ameliorant for improving crop production—a review. Biores. Technol..

[6] Lee H, Ha HS, Lee CH, Lee YB, Kim PJ (2006). Fly ash effect on improving soil properties and rice productivity in Korean paddy soils. Biores. Technol..

[7] Dhami NK, Mukherjee A, Reddy MS (2012). Improvement in strength properties of ash bricks by bacterial calcite. Ecol. Engin..

[8] Dhami NK, Mukherjee A, Reddy MS (2013). Viability of calcifying bacterial formulations in fly ash for applications in building materials. J.Ind. Microbiol. Biotechnol..

[9] Whiffin VS, van Paassen LA, Harkes MP (2007). Microbial carbonate precipitation as a soil improvement technique. Geomicrobiol. J..

[10] DeJong JT, Mortensen BM, Martinez BC, Nelson DC (2010). Bio-mediated soil improvement. Ecol. Engin..

[11] DeJong JT (2013). Biogeochemical processes and geotechnical applications: progress, opportunities and challenges. Géotechnique.

[12] Chu J, Stabnikov V, Ivanov V (2012). Microbially induced calcium carbonate precipitation on surface or in the bulk of soil. Geomicrobiol. J..

[13] Mujah D, Shahin MA, Cheng L (2016). State-of- the-art review of bio-cementation by microbially induced calcite precipitation (MICP) for soil stabilization. Geomicrobiol. J..

[14] Li M, Zhu X, Mukherjee A, Huang M, Achal V (2017). Biomineralization in metakaolin modified cement mortar to improve its strength with lowered cement content. J. Hazard. Mater..

[15] Li M, Fu Q, Zhang Q, Achal V, Kawasaki S (2015). Bio-grout based on microbially induced sand solidification by means of asparaginase activity. Sci. Rep..

[16] Cheng L, Cord-Ruwisch R (2012). *In situ* soil cementation with ureolytic bacteria by surface percolation. Ecol. Engin..

[17] Achal V, Mukherjee A, Basu PC, Reddy MS (2009). Strain improvement of *Sporosarcina pasteurii* for enhanced urease and calcite production. J. Ind. Microbiol. Biotechnol..

[18] ASTM D 698 - Standard Test Methods for Laboratory Compaction Characteristics of Soil Using Standard Effort (12,400 ft-lbs/ft^3^ (600 KN-m/m^3^)). ASTM International, West Conshohocken, PA (2012).

[19] ASTM D4318-00, Standard Test Methods for Liquid Limit, Plastic Limit, and Plasticity Index of Soils, ASTM International, West Conshohocken, PA (2000).

[20] ASTM D4546-14, Standard Test Methods for One-Dimensional Swell or Collapse of Soils, ASTM International, West Conshohocken, PA, (2014).

[21] Mishra AK, Dhawan S, Rao SM (2008). Analysis of swelling and shrinkage behavior of compacted clays. Geotech Geol Eng.

[22] Zaady E, Katra I, Barkai D, Knoll Y, Sarig S (2017). The coupling effects of using coal fly-ash and bio-inoculant for rehabilitation of disturbed biocrusts in active sand dunes. Land Degrad. Develop..

[23] Brooks MR (2009). Soil stabilization with fly ash and rice husk ash. Int J Res Rev Appl Sci.

[24] Singh SP, Roy N, Sangita S (2016). Strength and hydraulic conductivity of sedimented ash deposits treated with lime column. Inter. J. Geotech. Engin..

[25] Cheng L, Cord-Ruwisch R, Shahin MA (2013). Cementation of sand soil by microbially induced calcite precipitation at various degrees of saturation. Can Geotech J.

[26] Sham E (2013). 2013. Monitoring bacterially induced calcite precipitation in porous media using magnetic resonance imaging and flow measurements. J Contam Hydrol.

[27] Goodarzi AR, Akbari HR, Salimi M (2016). Enhanced stabilization of highly expansive clays by mixing cement and silica fume. Appl. Clay Science.

[28] Li, J., Wu, X. & Hou, L. Physical, mineralogical, and micromorphological properties of expansive soil treated at different temperature. *J*. *Nanomaterials* 848740, 10.1155/2014/848740 (2014).

[29] Lin B, Cerato AB (2012). Prediction of expansive soil swelling based on four micro-scale properties. Bull Eng Geol Environ.

[30] Seco A, Ramírez F, Miqueleiz L, García B (2011). Stabilization of expansive soils for use in construction. Appl. Clay Sci..

[31] Sharma NK, Swain SK, Sahoo UC (2012). Stabilization of a clayey soil with fly ash and lime: A micro level investigation. Geotech. Geolog. Engin..

