# Supplementary material for: Fly ash incorporated with biocement to improve strength of expansive soil
Source: Sci Rep. 2018 Feb 7;8:2565. doi: 10.1038/s41598-018-20921-0 (PMC5803229; doi:10.1038/s41598-018-20921-0)
Supplement: Supplementary file 1 — Supplementary information [file 41598_2018_20921_MOESM1_ESM.doc]

**SUPPLEMENTARY INFORMATION**

**Fly ash incorporated with biocement to improve strength of expansive soil**

**Mengmeng Li^1†^, Chaolin Fang^1†^, Satoru Kawasaki^2^, Varenyam Achal^1^***

**^1^**Shanghai Key Lab for Urban Ecological Processes and Eco-Restoration (SHUES), School of Ecological and Environmental Sciences, East China Normal University, Shanghai 200241, China

^2^Faculty of Engineering, Hokkaido University, Sapporo 0608628, Japan

†Joint first author


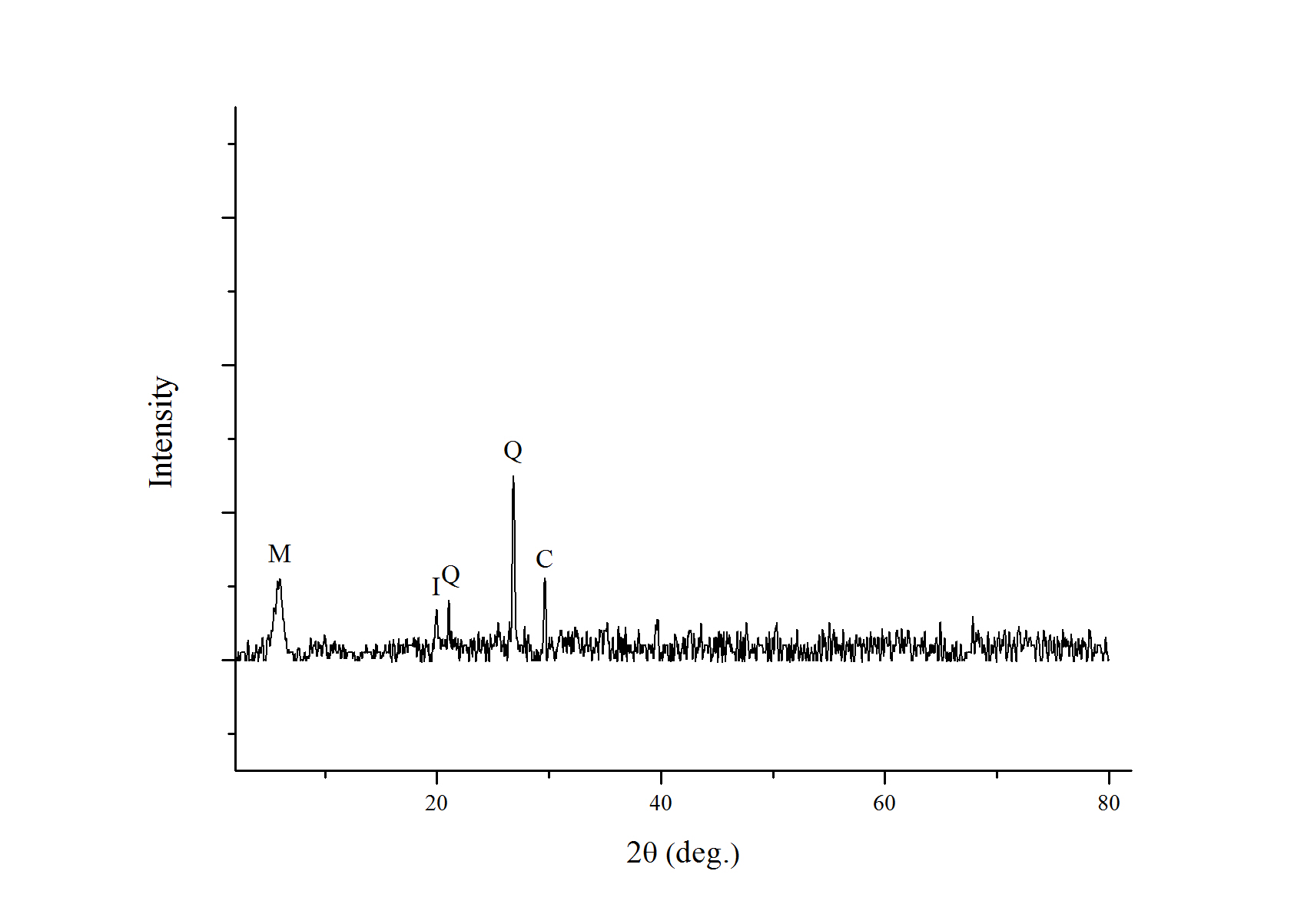


**Supplementary Figure 1** XRD spectra of expansive soil sample. [M: montmorillonite, I: illite, Q: quartz, C: calcite].
